# The Anti-Tumor Effect of HDAC Inhibition in a Human Pancreas Cancer Model Is Significantly Improved by the Simultaneous Inhibition of Cyclooxygenase 2

**DOI:** 10.1371/journal.pone.0075102

**Published:** 2013-09-11

**Authors:** Olivier Peulen, Arnaud Gonzalez, Paul Peixoto, Andrei Turtoi, Denis Mottet, Philippe Delvenne, Vincent Castronovo

**Affiliations:** 1 Metastasis Research Laboratory, GIGA-Cancer, University of Liege, Liege, Belgium; 2 Laboratory of Experimental Pathology, GIGA-Cancer, University of Liege, Liege, Belgium; Technische Universität München, Germany

## Abstract

Pancreatic ductal adenocarcinoma is the fourth leading cause of cancer death worldwide, with no satisfactory treatment to date. In this study, we tested whether the combined inhibition of cyclooxygenase-2 (COX-2) and class I histone deacetylase (HDAC) may results in a better control of pancreatic ductal adenocarcinoma. The impact of the concomitant HDAC and COX-2 inhibition on cell growth, apoptosis and cell cycle was assessed first *in vitro* on human pancreas BxPC-3, PANC-1 or CFPAC-1 cells treated with chemical inhibitors (SAHA, MS-275 and celecoxib) or HDAC1/2/3/7 siRNA. To test the potential antitumoral activity of this combination *in vivo*, we have developed and characterized, a refined chick chorioallantoic membrane tumor model that histologically and proteomically mimics human pancreatic ductal adenocarcinoma. The combination of HDAC1/3 and COX-2 inhibition significantly impaired proliferation of BxPC-3 cells *in vitro* and stalled entirely the BxPC-3 cells tumor growth onto the chorioallantoic membrane *in vivo*. The combination was more effective than either drug used alone. Consistently, we showed that both HDAC1 and HDAC3 inhibition induced the expression of COX-2 via the NF-kB pathway. Our data demonstrate, for the first time in a Pancreatic Ductal Adenocarcinoma (PDAC) model, a significant action of HDAC and COX-2 inhibitors on cancer cell growth, which sets the basis for the development of potentially effective new combinatory therapies for pancreatic ductal adenocarcinoma patients.

## Introduction

Pancreatic ductal adenocarcinoma (PDAC) lists among the most deadly form of cancers [1]. Early-stage of the disease is clinically silent and the diagnosis of the disease is mostly made at an advanced stage. This late diagnosis contributes to one of the lowest 5-year survival rate (only 3%) [2]. Today PDAC are treated by surgery and/or adjuvant therapy with gemcitabine, increasing only slightly the median survival of the patients. There is therefore an urgent need to develop new effective therapies for PDAC patients.

There are abundant evidence indicating that deregulation of histone acetylation contributes to pancreas cancer development and progression [3]. Histone deacetylases (HDAC) represent a family of enzymes that regulate paramount cellular activities including epigenetic silencing of tumor suppressor genes and modulation of protein functions. We and others have shown that HDAC inhibition exerts both anti-cancer and anti-angiogenesis activities [4]–[6]. HDAC expression is altered in PDAC, including HDAC1, HDAC2, HDAC3 and HDAC7 [7]–[10]. Preclinical studies have suggested that HDAC inhibition hold significant potential for the development of new anticancer therapies [11]. Accordingly, several HDAC inhibitors have been recently approved by the Food and Drug Administration for the treatment of Cutaneous T-Cell Lymphoma while new molecules are currently in phase III clinical trials. However, when used in monotherapy, HDAC inhibitors showed limited efficacy in various solid malignancies, including PDAC [3], [12], [13]. Indeed, LAQ824 or MS-275 have been evaluated in phase I clinical trials in solid cancers, including PDAC, without any objective clinical response [14], [15]. Alternatively, HDAC inhibitors have been used in combined therapy strategies [16], [17], with some combinations generating promising effects for human PDAC *in vitro* [18]–[21] or in experimental tumors [22]. Unfortunately, these results do not translate in clinical trials [23], [24].

The lack of efficacy of HDAC inhibitors in pancreatic cancer could be linked to the pleiotropic activities of HDACs in cell biology [25], [26] leading to undesired pro-cancer effects. For example, a recent study demonstrated that pan-HDAC inhibitors induce cyclooxygenase-2 (COX-2) expression in lung cancer cells, leading to a stimulation of endothelial cell proliferation [27]. Since COX-2 has been also associated to pancreatic cancer cell proliferation [28] or tumor growth [29]–[31], we hypothesized that COX-2 overexpression may also be induced in PDAC when treated with HDAC inhibitors, leading to reduced efficiency and hence therapeutic failure.

To test the biological relevance of combining class I HDAC and COX-2 inhibitors *in vivo*, we devised a refined PDAC chick chorioallantoic membrane (CAM) model based on our previous work [32]. The CAM model has been successfully used with several cell lines to produce tumors [33], [34]. Similarly to the murine model, most steps of tumor progression are recapitulated in a very short period of time [35]. Previously, BxPC-3 pancreatic cancer cells were already demonstrated to produce vascularized 100 µm long tumor nodes on CAM [32]. However, the small size of the nodules represented a significant limitation for structural observation, accurate volume evaluation and study of drug efficacy. Here, we have established and implemented a refined BxPC-3 PDAC model featuring a dramatic increase (64-fold) in tumor size and displaying structural architecture and protein expression mimicking human PDAC. This model was successfully exploited to demonstrate that the combination of class I HDAC and COX-2 inhibitors result in a complete tumor growth inhibition.

## Materials and Methods

### Cells and chemicals

BxPC-3 (ATCC CRL-1687), PANC-1 (ATCC CRL-1469) and CFPAC-1 (ATCC CRL-1918) are human pancreatic cancer cell lines derived respectively from PDAC [36], pancreas duct epithelioid carcinoma [37] and PDAC liver metastasis [38]. BxPC-3 were a generous gift from Prof. Bikfalvi (Inserm u1029, Bordeaux, France), Panc-1 were a generous gift from Prof. Muller and Burtea (NMR Laboratory, University of Mons, Belgium). CFPAC-1 were bought from ATCC. Celecoxib was obtained from the University Pharmacy (Kemprotec Ltd, Middlesbrough, UK). MS-275 and SAHA were purchased from Enzo Life Sciences (Antwerpen, Belgium). Other chemicals were purchased from Sigma (Bornem, Belgium).

### Cell culture

BxPC-3 human pancreatic cancer cell line were maintained in RPMI1640 medium supplemented with glucose (2.5 g/L), sodium pyruvate (1 mM) and FBS (10%). PANC-1 were maintained in DMEM supplemented with FBS (10%). CFPAC-1 were maintained in Iscove's Modified Dulbecco's Medium with FBS (10%). Cells were treated with MS-275, celecoxib or combination of both as well as with suberoylanilide hydroxamic acid (SAHA) solubilized in medium with 0.1% DMSO.

### Small interfering RNA transfection

HDAC-specific small interfering RNA (siRNA) were synthesized by Eurogentec (Seraing, Belgium). NF-kB p65 SMARTpool siRNA were bought from Thermo Fisher-Dharmacon (Whaltham, MA). Lipofectamine-mediated transfections were performed at a siRNA concentration of 40 nM following manufacturer’s recommendations (Life Technologies, Carlsbad, NM). GL3 was an irrelevant siRNA targeting luciferase. siRNA sequences were published previously [5].

### Cell growth

Equal densities of cells were seeded in complete medium and were harvested at the indicated time-points. The cell numbers were indirectly determined using Hoechst incorporation. Results were expressed as DNA content.

### Western-blotting

BxPC-3 cells or frozen tumors were disrupted in lysis buffer (1% SDS, 40 mM Tris-HCl pH7.5) in the presence of protease and phosphatase inhibitors. Proteins were separated by SDS-PAGE (6–12.5%) then electrotransfered on nitrocellulose membranes. Following primary antibodies were used: anti-COX-2 (Cayman Chemicals, Ann Arbor, MI), anti-HDAC1 (Cell Signalling, Danvers, MA), anti-HDAC2 (Santa Cruz Biotechnology, Santa Cruz, CA), anti-HDAC3 (Cell Signalling, Danvers, MA), anti-acetylated-Histone-3 (Millipore, Billerica, MA), anti-HDAC7 (Santa Cruz Biotechnology, Santa Cruz, CA), anti-phospho-IkBα (Cell Signalling, Danvers, MA), anti-p65 (Cell signaling, Danvers, MA), anti-p21 (Santa Cruz Biotechnology, Santa Cruz, CA), anti-p27 (BD Biosciences, Franklin Lakes, NJ), anti-pRB (BD Biosciences, Franklin Lakes, NJ), anti-E2F1 (Santa Cruz Biotechnology, Santa Cruz, CA), anti-MEK2 (Cell signaling, Danvers, MA), anti-ORC2 (Cell signaling, Danvers, MA), anti-caspase-3 (Cell Signalling, Danvers, MA) and anti-HSC70 (Santa Cruz Biotechnology, Santa Cruz, CA). Immunodetection was performed using appropriate secondary antibody conjugated with horseradish peroxidase.

### Quantitative real-time RT-PCR

Total RNA extraction and quantitative real-time RT-PCR were performed as previously described [39]. Human COX-2 expression was detected using a commercial RT-qPCR TaqMan assay (Hs00153133-m1; Applied Biosystems, Carlsbad, NM). Human IL-8 expression was detected using specific forward (5′-GAAGGAACCATCTCACTGTGTGTAA-3′) and reverse (5′-ATCAGGAAGGCTGCCAAGAG-3′) primers synthesized by Eurogentec (Seraing, Belgium).

### Annexin V/propidium iodide staining

Apoptotic cells were determined by annexin V-FITC and non-vital dye propidium iodide (PI) staining with a FITC-Annexin V apoptosis detection kit I (BD Biosciences, Franklin Lakes, NJ) according to the manufacturer’s instructions. Flow cytometry was performed on a FACSCalibur II™ and samples were analyzed using CellQuest™ software (BD Biosciences, Franklin Lakes, NJ).

### Cell cycle analysis

The relative percentage of cells in each stage of the cell cycle was analyzed as previously described [33] by flow cytometric analysis with FACSCalibur II™ and ModFit LT™program.

### Tumor growth on CAM

Fertilized chicken eggs were opened as previously described [32]. On post-fertilization day 11, CAM surface was gently scratched with a needle and 3.5×10^6^ BxPC-3, PANC-1 or CFPAC-1 cells in suspension with 50% matrigel in a final volume of 100 µL were grafted on the CAM enclosed by a 6-mm plastic ring. The implantation day was considered as day 0 of tumor development. Drugs (celecoxib 8 µM and/or MS-275 0.2 µM in a 30 µl final volume) were applied daily directly on tumor starting at day 2. At day 7, the tumors were excised from the CAM and digital pictures were taken using a stereomicroscope. Tumor volume was calculated using an ellipsoid formula: Volume = (4×πxZ_1_×Z_2_×Z_3_)/3 where Z_1−3_ are the main radius of the tumor.

### Ethics statement

All animal experiments were approved by the Animal Welfare Committee of the University of Liège (approval #1278).

### Histology procedure

BxPC-3 tumors were washed in PBS and then fixed in 4% paraformaldehyde for 30min at 4°C. The tumors were embedded in paraffin and 5 µm sections were stained with Hematoxylin-eosin or Masson’s trichrome.

Immunoperoxydase and amylase-periodic acid Schiff (PAS) staining were performed on 5 µm sections, respectively, with the BenchMark XT IHC/ISH automated stainer and the NexES Special Stains (Ventana Medical Systems Inc, Tucson, AZ) according to the manufacturer’s instructions. Following antibodies were used: anti-cytokeratin 7 (CK7 - Dako, Glostrup, Denmark), anti-cytokeratin 19 (CK19 - Roche Diagnostics, Vilvoorde, Belgium), anti-cytokeratin 20 (CK20 - Dako, Glostrup, Denmark), anti-CD56 (Novocastra, Leica Microsystem Inc, Buffalo Grove, IL), anti-carcinoembryonic antigen (CEA - Roche Diagnostics, Vilvoorde, Belgium), anti-Ki67 (Dako, Glostrup, Denmark), anti-latent transforming growth factor-beta binding protein 2 (LTBP2 – Santa Cruz Biotchnology, Santa Cruz, CA), anti-transforming growth factor beta-induced (TGFBI - Cell Signalling, Danvers, MA), anti-myoferlin (Sigma, Bornem, Belgium) and anti-desmin (Dako, Glostrup, Denmark) were used for the primary reaction.

Ki67 quantification was performed on randomly taken pictures (3 pictures from each tumor, 3 tumors in each experimental group). After channel splitting, blue channel pictures were binarized according to the brightness. The size of the area occupied by all cells or by Ki67-positive cells was measured using imageJ 1.46r software.

In order to visualize the tumor vasculature, thick rehydrated tissue sections (35 µm) were incubated for 30min in the dark with 0.05% Triton X-100 in PBS containing 5 µg/mL *Sambucus nigra* agglutinin (SNA, Vector Laboratories, Burlingame, CA). The sections were washed with 0.05% Triton X-100 in PBS and visualized with confocal microscope (Leica SP2). Three-dimensional images were reconstructed with Imaris software (Bitplane Scientific Software, Zurich, Switzerland).

### Statistical analysis

All results were reported as means with standard deviation. Statistical analysis was performed using one-way or two-way ANOVA depending on the number of grouping factors. Group means were compared by a Bonferroni's post-test. P<.05 was considered as statistically significant. All experiments were performed as 3 independent biological replicates.

## Results

### Class I HDAC inhibition reduced pancreas cancer cell growth in vitro

**Figure 1:**
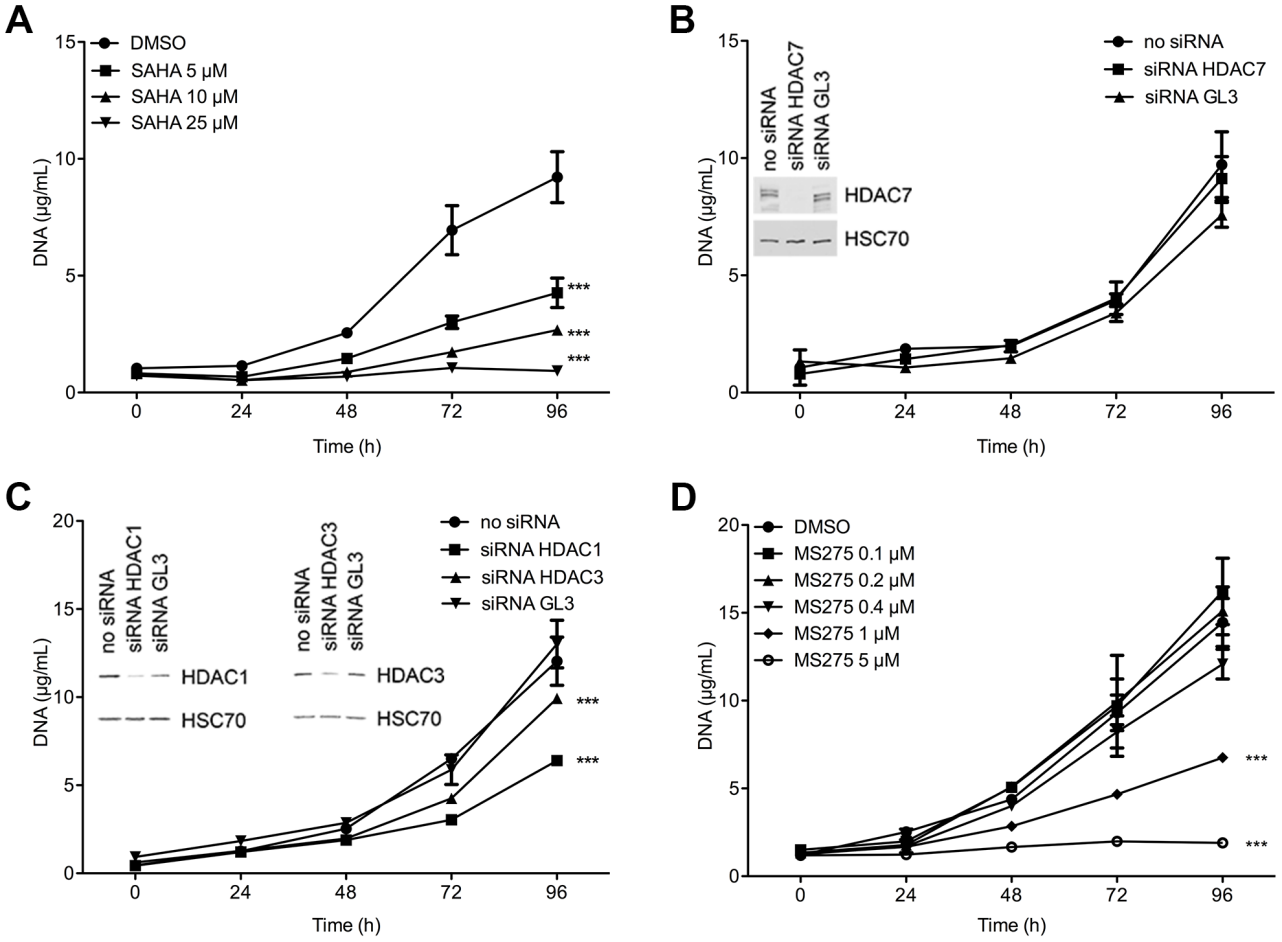
Effect of HDAC silencing or inhibition on BxPC-3 cell proliferation. (A) Time-dependent and dose-dependent effects of SAHA on cell proliferation. (B) Time-dependent effect of class IIa HDAC7 silencing on cell proliferation. HDAC7 expression was detected by western-blot 48h after siRNA transfection. HSC70 was used as a loading control. (C) Time-dependent effect of class I HDAC1 or –3 silencing on cell proliferation.. HDAC1 and HDAC3 expression was detected by western-blot 48h after siRNA transfection. HSC70 was used as a loading control. (D) Time-dependent and dose-dependent effects of MS-275 on cell proliferation ***P<.001 versus DMSO or GL3 conditions. Results are expressed as mean ± s.d., n≥3 in each condition.

BxPC-3 cells have been described to express altered levels of class I HDAC1, HDAC3 and class II HDAC7 [40], [41]. To evaluate the role of these HDAC in BxPC-3 cells, we first examined their time-dependent and concentration-dependent growth in presence of SAHA, a class I/II inhibitor (Figure 1A). Our results confirmed that BxPC-3 cells were sensitive to SAHA, with a 50% growth reduction (P<.001) observed at 5 µM. Next, we selectively silenced HDAC1, –3 or –7 using siRNA to examine the individual involvement of these HDAC in the SAHA-induced growth reduction. HDAC7 silencing did not affect cell growth (Figure 1B). However, HDAC1 and HDAC3 silencing reduced significantly BxPC-3 cell growth by respectively 50% (P<.001) and 20% (P<.001) (Figure 1C). In order to evaluate this decrease in cell growth with clinically compatible drug, we evaluated the time-dependent and concentration-dependent growth of BxPC-3 cells in presence of MS-275 (HDAC1 and HDAC3 inhibitor). MS-275 (1 µM) reduced BxPC-3 cell growth by 50% (P<.001) whereas 5 µM abolished completely the growth (P<.001) (Figure 1D).

### Class I HDAC inhibition induced COX-2 expression in vitro

**Figure 2:**
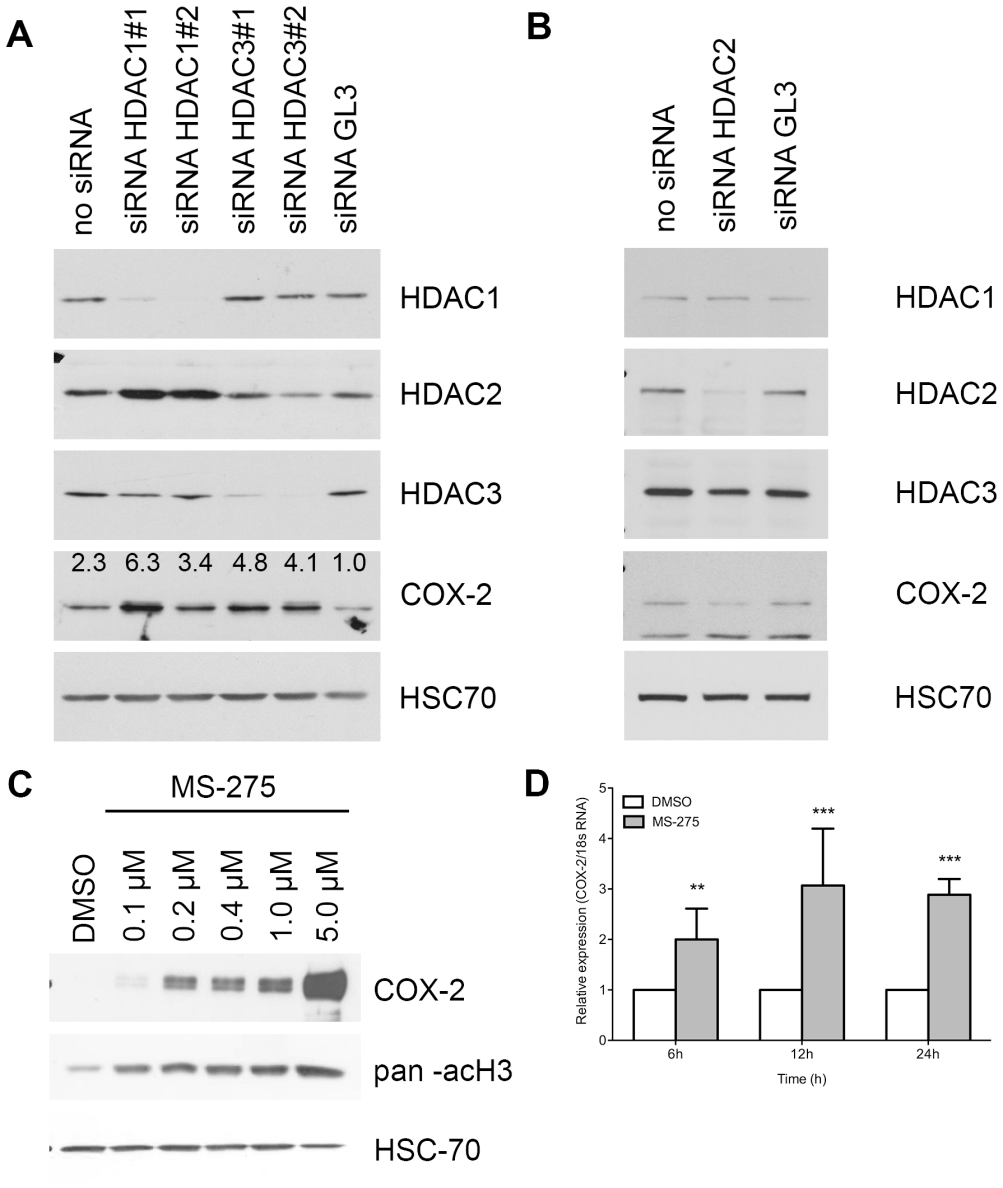
Effect of HDAC silencing or inhibition on COX-2 expression in BxPC-3 cells. (A) Western-blot detection of COX-2 and HDAC in 20 µg BxPC-3 proteins 48h after HDAC1 or HDAC3 siRNA transfection. (B) Western-blot detection of COX-2 and HDAC in 20 µg BxPC-3 proteins 48h after HDAC2 siRNA transfection. (C) Dose-dependent effects of 48h MS-275 treatment on COX-2 expression. Acetylated-histone H3 was used as a control of treatment efficacy. HSC70 was used as a loading control. (D) Time-dependent relative expression of COX-2 mRNA in BxPC-3 cells treated with 1 µM MS-275. Results are expressed as mean ± s.d., n = 3.

The limited efficiency of HDAC inhibitors in clinical trials including PDAC patients could be explained, at least in part, by the potential up regulation of the expression of COX-2 in pancreatic malignant cells. To evaluate this hypothesis, we first analyzed COX-2 expression in BxPC-3 cells silenced for HDAC1, HDAC2, HDAC3 or treated with MS-275. HDAC1 or HDAC3 repression induced respectively a 6.3-fold and a 4.8-fold increase of COX-2 expression at protein level (Figure 2A) while HDAC2 silencing reduced COX-2 expression (Figure 2B). HDAC1 silencing induced an HDAC2 overexpression.

Treatment of BxPC-3 cells with MS-275 showed similar effects on COX-2 accumulation in a concentration-depend manner (Figure 2C). To determine whether COX-2 induction occurs at transcriptional level, we analyzed COX-2 mRNA level by RT-qPCR following 6, 12, and 24h of MS-275 treatment. We found that COX-2 gene expression was up-regulated following the MS-275 treatment in a time-dependent manner (Figure 2D).

**Figure 3:**
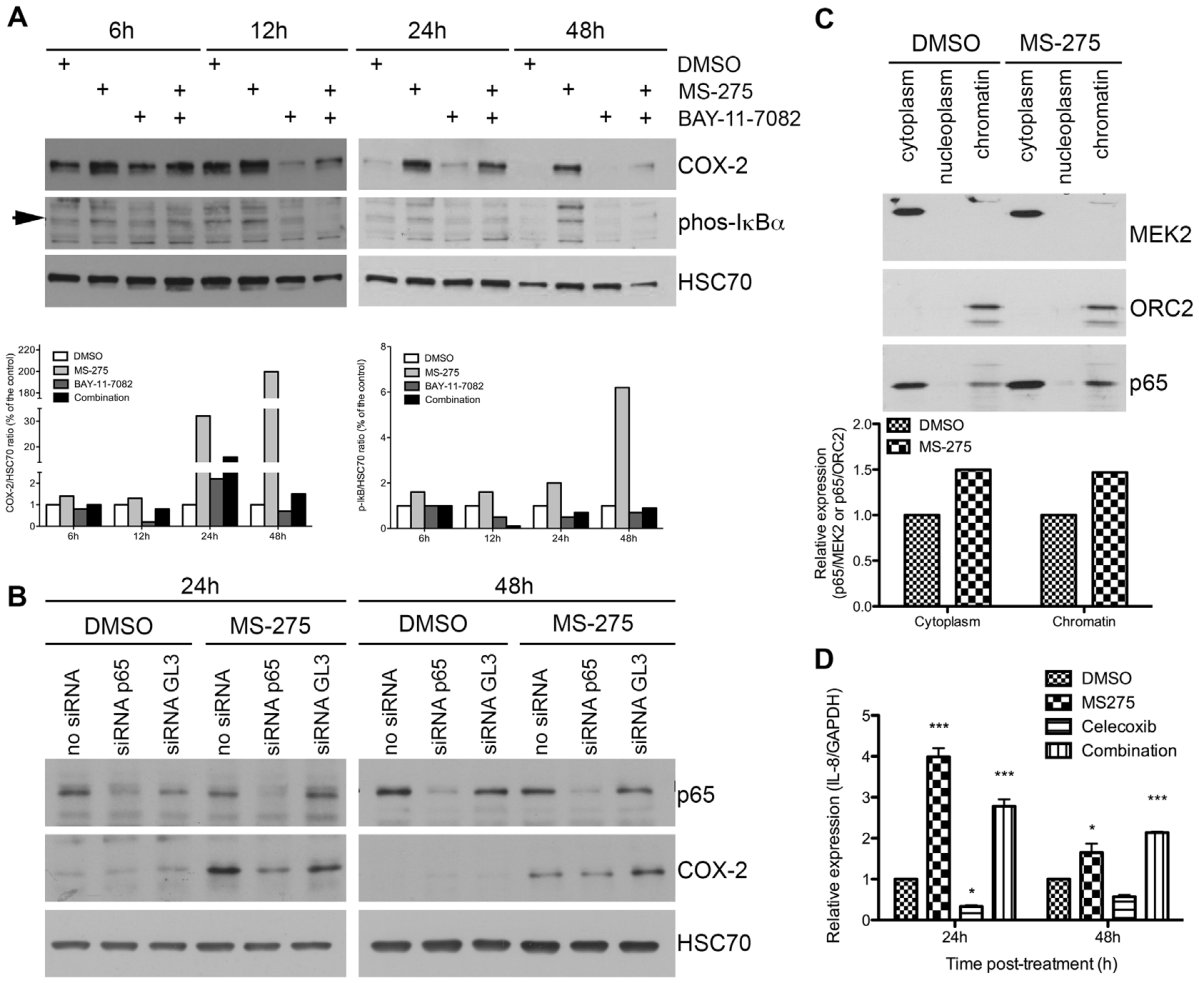
Effect of HDAC inhibition on NF-kB activation in BxPC-3 cells. (A) Effect of an IKK inhibitor (10 µM BAY-11-7082) on 1 µM MS-275-induced COX-2 expression. Phospho-IkBα was used as a control of BAY-11-7082 treatment efficacy. HSC70 was used as a loading control. Densitometry was expressed as a COX-2/HSC70 or IkBα/HSC70 ratio. (B) Western-blot detection of COX-2 in 20 µg BxPC-3 proteins after 1 µM MS-275 treatment and p65 siRNA transfection. HSC70 was used as a loading control. (C) Western-blot detection of p65 in 15 µg BxPC-3 cytoplasm, nucleoplasm or chromatin-associated proteins after 1 µM MS-275 treatment. MEK2 and ORC2 were used as a loading control respectively in cytoplasm and chromatin fractions. Densitometry was expressed as a p65/MEK2 or p65/ORC2 ratio. (D) Time-dependent relative expression of IL-8 mRNA in BxPC-3 cells treated with 1 µM MS-275, 10 µM Celecoxib or a combination of the drugs. Results are expressed as mean ± s.d. ***P<.001, *P<.05 versus DMSO. n≥3 in each condition.

To study the mechanisms by which class I HDAC inhibition induces COX-2, we explored the known link between NF-kB and HDAC1/3 [42], [43] and tested the possibility that MS-275-induced COX-2 expression could be NF-kB dependent. Accordingly, we co-treated cells with MS-275 and BAY-11-7082, an IkBα kinase (IKK) inhibitor. BAY-11-7082 reduced by 30% to 90% the COX-2 expression following respectively 6h to 48h of MS-275 treatment (Figure 3A), suggesting the MS-275-induced expression of COX-2 is, at least in part, NF-kB dependent. This hypothesis was supported by p65-silencing and p65 translocation to the nucleus. COX-2 expression was induced by a 24h treatment with MS-275 and was prevented by p65 siRNA (Figure 3B). Moreover, 24h MS-275 treatment induced an increase by 50% of the p65 protein level in the cytoplasm and in the chromatin fraction of BxPC-3 cells (Figure 3C). The same MS-275 treatment induced the gene expression of IL-8 (Figure 3D), a direct target of NF-kB.

### Combined inhibition of class I HDAC and COX-2 inhibits cell growth in vitro

**Figure 4:**
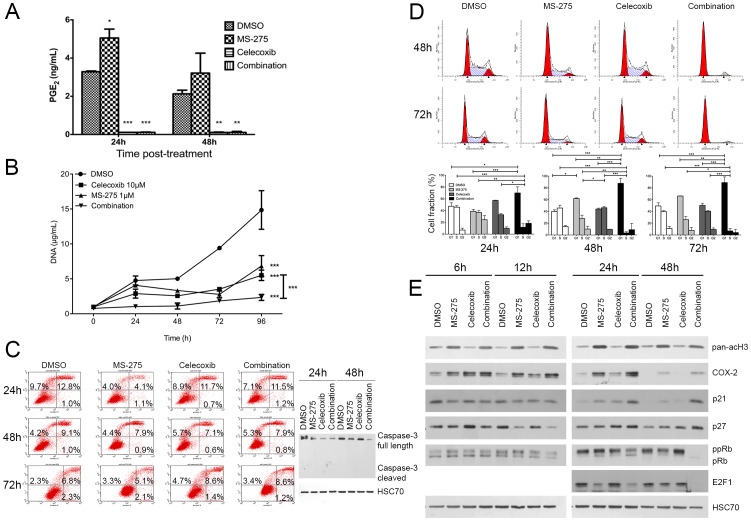
Effect of HDAC and COX-2 coinhibition in BxPC-3 cells. (A) ELISA assay of PGE_2_ in cell culture media 24h and 48h after 1 µM MS-275 and 10 µM celecoxib treatment. (B) Time-dependent effects of MS-275 and celecoxib on cell growth. (C) Time-dependent effects of 1 µM MS-275 and 10 µM celecoxib on apoptotic cell ratio by annexin V/PI flow cytometry and on caspase-3 cleavage. (D) Time-dependent effects of 1 µM MS-275 and 10 µM celecoxib on cell cycle by PI incorporation. (E) Western-blot detection of p21, p27, pRb ppRb and E2F1 in 20 µg BxPC-3 proteins 6 to 48h after 1 µM MS-275 and 10 µM celecoxib treatment. HSC70 was used as a loading control. Results are expressed as mean ± s.d., ***P<.001, **P<.01, *P<.05 versus DMSO or indicated conditions. n≥3 in each condition.

In order to validate our hypothesis that class I HDAC inhibition mediated induction of COX-2 might contribute to the low efficiency of HDAC based therapy in PDAC patients, we have combined the latter with celecoxib, a selective COX-2 inhibitor at IC50 (respectively 1 µM of MS-275 and 10 µM of celecoxib). The MS-275-induced COX-2 overexpression led to a 50% increase of PGE2 concentration in the culture media (Figure 4A). BxPC-3 cell treatment with celecoxib alone or in combination with MS-275 reduced significantly the PGE_2_ concentration in the cell media. We then analyzed the impact of these treatments on the cell growth. The combination of the two drugs reduced significantly (>85%, P<.001) the BxPC-3 cell growth in comparison with using either drug alone (Figure 4B). We next asked the question whether this reduction is due to induction of apoptosis and performed an annexin V/propidium iodide staining at 24, 48 and 72h (Figure 4C) following the treatment. None of the individual drugs nor their combination were able to induce apoptosis. These results were confirmed by western-blot, showing intact caspase-3 in all samples (Figure 4C). To further investigate the mechanisms of the observed cell growth arrest, we next examined the effect of MS-275/celecoxib combination on the cell cycle (Figure 4D). MS-275 alone, but not celecoxib, increased the proportion of cell in G1 by 50% at 48h. However, MS-275/celecoxib combination decreased significantly (P<.001) the proportion of cells in S phase at 24 (–74%), 48 (–92%) and 72h (–82%) and increased significantly (P<.001) the proportion in G1 phase at 24 (+48%), 48 (+119%) and 72h (+80%). To validate these results we analyzed by western blot the expression of cell cycle markers and found a clear accumulation of p21^WAF1^ and p27^Kip1^, two cell cycle inhibitors, at 24h and 48h after the co-administration of MS-275 and celecoxib (Figure 4E). Consistently, the hyperphosphorylated form of pRb was less abundant when BxPC-3 cells were co-treated with MS-275/celecoxib. The hypophosphorylated form of pRb appeared with the co-inhibition of class I HDAC and COX-2. The whole pRb protein disappeared at 48h after the cotreatment. This disappearance was already observed by others after a p21^WAF1^ or p27^Kip1^ accumulation [44]. The E2F1 transcription factor, a S-phase orchestrator, became undetectable 48h after co-administration of MS-275 and celecoxib. These results show that cellular growth inhibition is associated to a G0/G1 phase blockage.

BxPC-3 is a PDAC cell line characterized by its KRAS wildtype, while mutations of the gene coding for this protein is the most common genetic alteration observed in human PDAC. However, BxPC-3 cells overexpress COX-2, a situation noted in 50% of human PDAC. We have decided to extend our observations regarding the interest of the combined treatment in pancreatic cancer by examining the efficiency of such combined treatment on two human pancreas cell lines with reported KRAS mutations. The first cell line was PANC-1 ([12 ASP]-KRAS) in which COX-2 was undetected at the protein level [45]. The second cell line was CFPAC-1 ([12 VAL]-KRAS) but in which COX-2 was detected at protein level [45].

**Figure 5:**
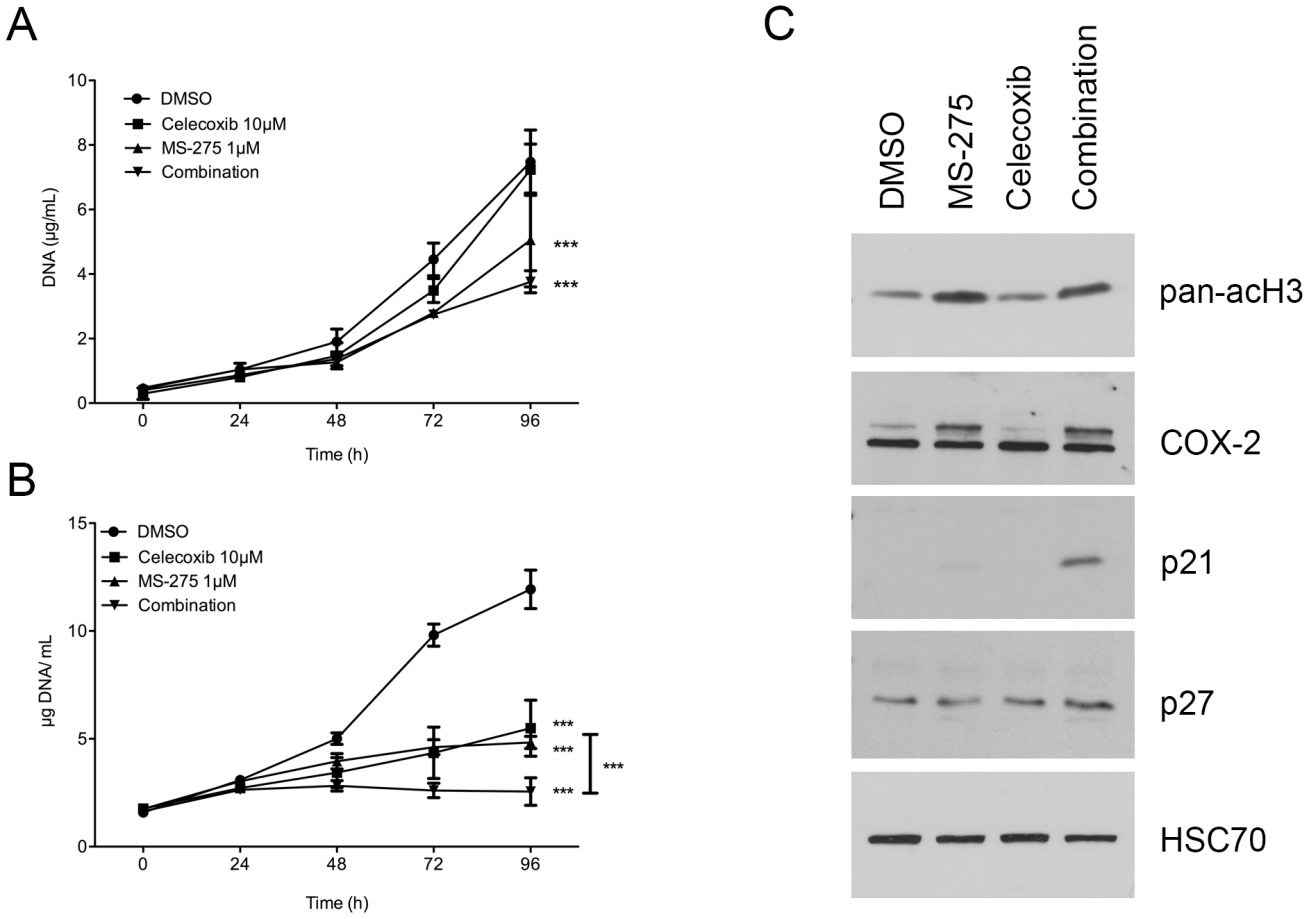
Effect of HDAC and COX-2 coinhibition in PANC-1 and CFPAC-1 cells. (A) Time-dependent effects of MS-275 and celecoxib on PANC-1 cell growth. (B) Time-dependent effects of MS-275 and celecoxib on CFPAC-1 cell growth. (C) Western-blot detection of Cox-2, p21, p27 in 30 µg CFPAC-1 proteins 48h after 1 µM MS-275 and 10 µM celecoxib treatment. HSC70 was used as a loading control. Results are expressed as mean ± s.d., ***P<.001 versus DMSO or indicated conditions. n≥3 in each condition.

PANC-1 cell line was cultured with MS-275, celecoxib or both drugs in combination. Celecoxib 10 µM did not alter cell growth when MS-275 1 µM reduced significantly (p<,001) cell growth by 32%. The combination of the two drugs reduced the PANC-1 cell growth (49%, P<.001). However, the combination-induced growth inhibition was not significantly different from the MS-275-induced one (Figure 5A). In this cell line, MS-275 did not induce the expression of COX-2 (data not shown).

CFPAC-1 cell line was cultured in the same conditions. Celecoxib 10 µM reduced cell growth by 54% (p<,001) and MS-275 1 µM reduced cell growth by 59% (p>,001). Here, the combination of the two drugs reduced significantly (79%, P<.001) CFPAC-1 cell growth in comparison to either drug alone (Figure 5B). We then analyzed by western blot the expression of COX-2 and cell cycle markers in CFPAC-1 cells 48h after drugs administration. We showed an MS-275-induced accumulation of COX-2 like in BxPC-3 cells (Figure 5C). We found also an accumulation of p21^WAF1^ and p27^Kip1^ after the co-administration of MS-275 and celecoxib (Figure 5C), suggesting a cell cycle arrest.

### BxPC-3 CAM tumor mimics human PDAC

**Figure 6:**
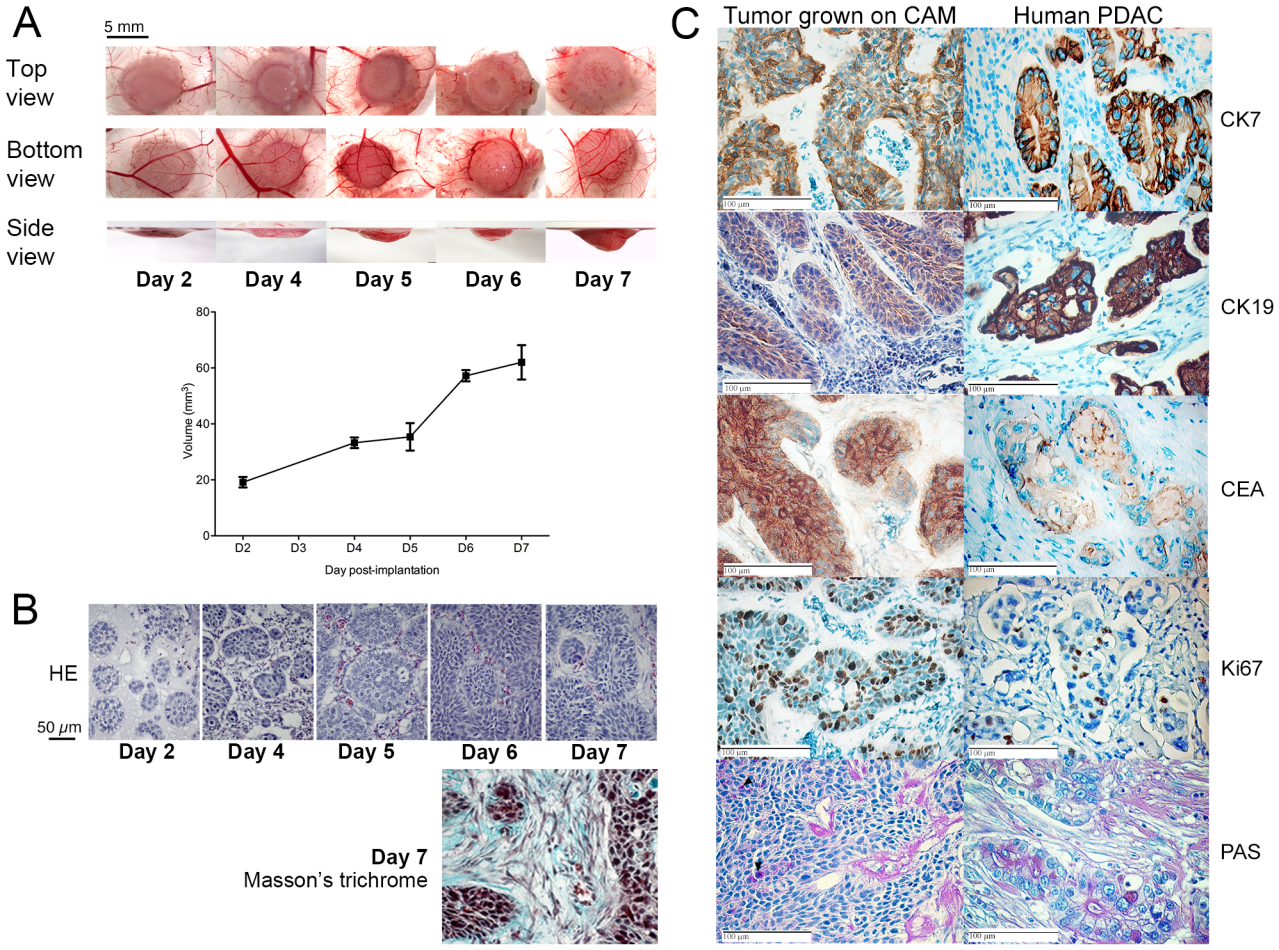
Growth curve and immunohistologic characterization of BxPC-3 tumors grown on CAM. (A) Cells were implanted on CAM at embryonic day 11 and collected 2, 4, 5, 6 or 7 days after implantation. Macroscopic pictures were obtained at the same magnification from top, bottom and side view. Results are expressed as mean ± s.d., n>5 at each time-point. (B) Histologic (Haematoxylin-Eosin or Masson’s trichrome staining) analysis of tumors collected 2, 4, 5, 6 or 7 days after implantation. (C) Immunohistology of tumors 7 days after BxPC-3 implantation on CAM and human PDAC tumors. CK7  =  Cytokeratin-7, CK19  =  cytokeratin-19, CEA  =  Carcinoembryonic antigen, PAS  =  Amylase-periodic acid Schiff staining.

The evaluation of new drugs or drug combinations for pancreas cancer will be eased by the availability of easy, ethically and economically sustainable animal models. Thus, we have undertaken to refine a human pancreas chorioallantoic membrane (CAM) model based on our initial work [32]. Embedding BxPC-3 cells into matrigel prior to CAM implantation generated a major improvement in the tumor volume. Indeed, following implantation, the tumor volume increased linearly (r^2^ = 0.87) until day 7 (Figure 6A). At the time of tumor collection (day 7), an average tumor volume of 59.95±15.34 mm^3^ (n = 10) was observed. BxPC-3 CAM tumors grew inside the CAM connective tissue as a unique spheric nodule. The same procedure was followed for BxPC-3, PANC-1 and CFPAC-1 cell lines. PANC-1 did not grow on CAM when CFPAC-1 grew as very small nodules (1 mm long).

BxPC-3 CAM tumor histology (Figure 6B) revealed large islets of cohesive cells, some of which showed a nascent central lumen and were isolated from each other by a collagen-containing extracellular matrix with several sparse fibroblast-like cells demonstrating the presence of an interstitial stroma.

To further validate our human pancreas cancer CAM model, we compared the expression of the cytokeratin-7, -19, -20, CD56, CEA and Ki67 using immunohistochemistry to human PDAC. We also checked for mucin and proteoglycan production utilizing the PAS staining. Tumoral cells from both BxPC-3 CAM tumor and PDAC samples were strongly positive for cytokeratin-7 and -19, CEA and Ki67 (Figure 6C) but negative for cytokeratin-20 and CD56 (data not shown). Both tumors were positive for PAS staining. Altogether, the data showed remarkable histology and biomarker expression similarities between the BxPC-3 CAM model and PDAC from human patients.

**Figure 7:**
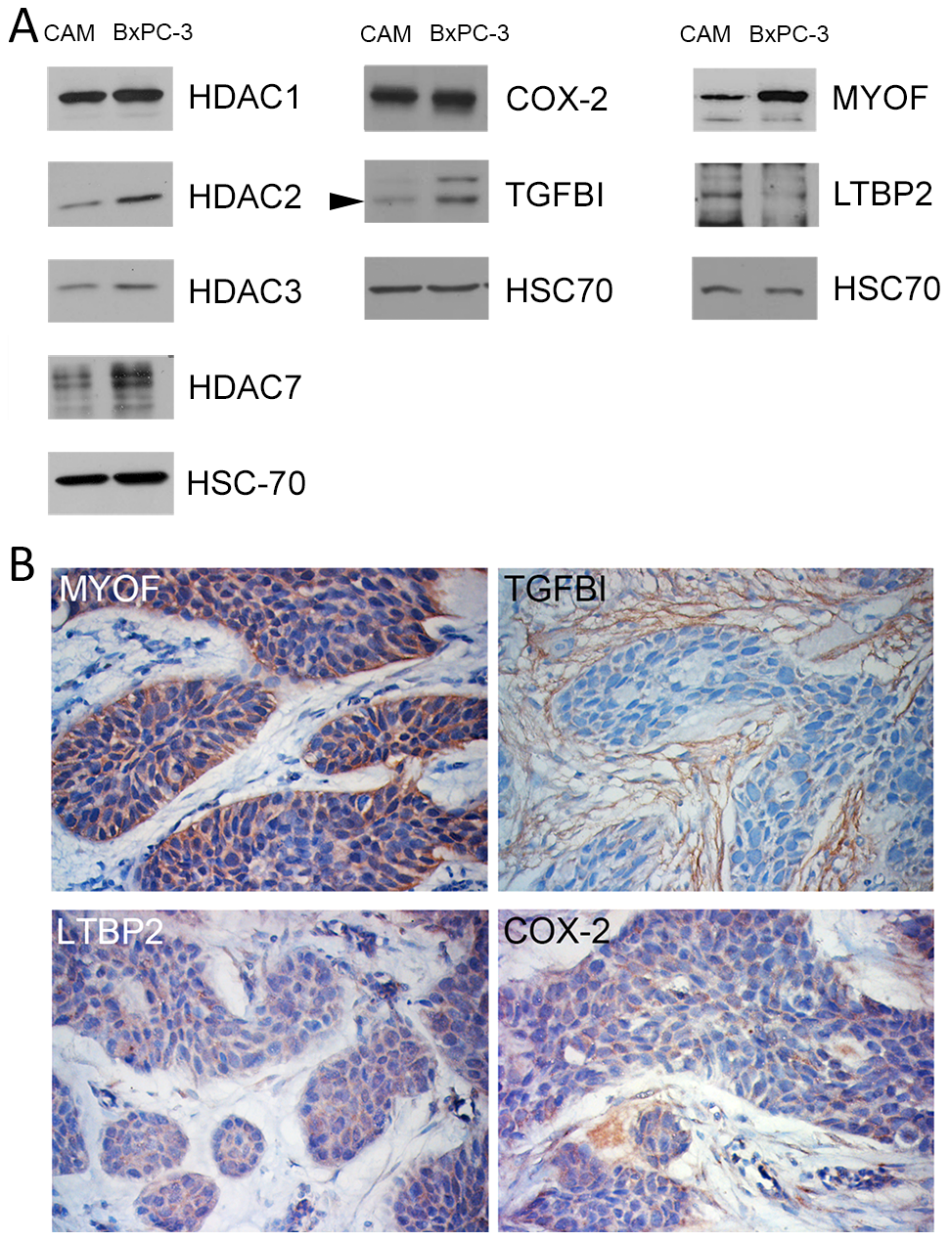
Biomarker detection in tumors 7 days after BxPC-3 implantation on CAM. (A) Western-blot detection of HDAC1, HDAC2, HDAC3, HDAC7, COX-2, TGFBI, MYOF, LTBP2 in 20 µg PDAC-CAM or BxPC-3 proteins. HSC70 was used as a loading control. (B) Immunoperoxydase labelling of MYOF, TGFBI, LTBP2, COX-2.

Furthermore, our recent work on targetable biomarkers in human PDAC [46] identified several biomarker candidates among which myoferlin, transforming growth factor beta-induced and latent-transforming growth factor beta-binding protein 2. Immunohistochemistry and western-blot confirmed the presence of these new PDAC biomarkers in the BxPC-3 CAM tumors (Figure 7A–B). Finally**,** using western blot we confirmed that HDAC1, HDAC2, HDAC3 and COX-2 are expressed in the BxPC-3 CAM tumor (Figure 7A).

**Figure 8:**
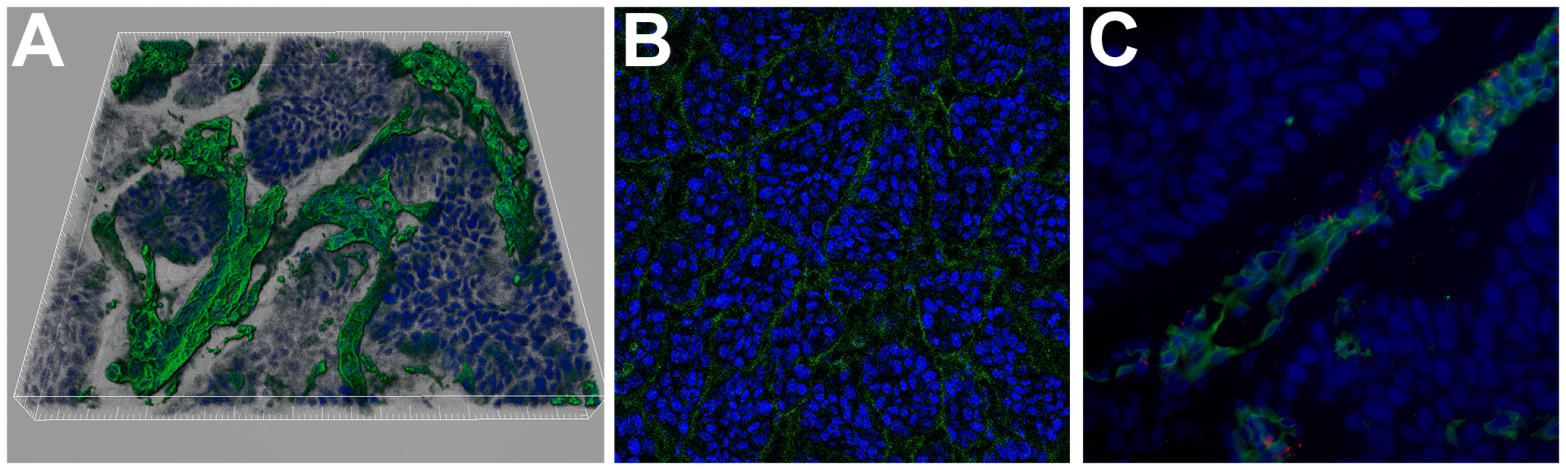
Blood vessel detection in tumors 7 days after BxPC-3 implantation on CAM. (A) Imaris 3D reconstruction from a 35 µm stacked image after SNA staining (green). Nuclei were counter stained with DAPI (blue). (B) Confocal image after FITC (green) injection in CAM blood vessels. Nuclei were counter stained with TOPRO (blue) (C) Desmin immunodetection (red) in PDAC-CAM stained with SNA (green). Nuclei were counter stained with DAPI (blue).

We next demonstrated that tumors were functionally vascularized. BxPC-3 CAM blood vessels were stained by FITC-conjugated SNA and 3D reconstructed after confocal acquisition. BxPC-3 CAM tumors displayed blood vessels around pancreatic islets (Figure 8A). The fluorescence of tumor stroma after fluorescent dye injection in the CAM vasculature confirms that the vessels are functional (Figure 8B) and the detection of desmin positive pericytes suggests vessel stabilization (Figure 8C).

**Figure 9:**
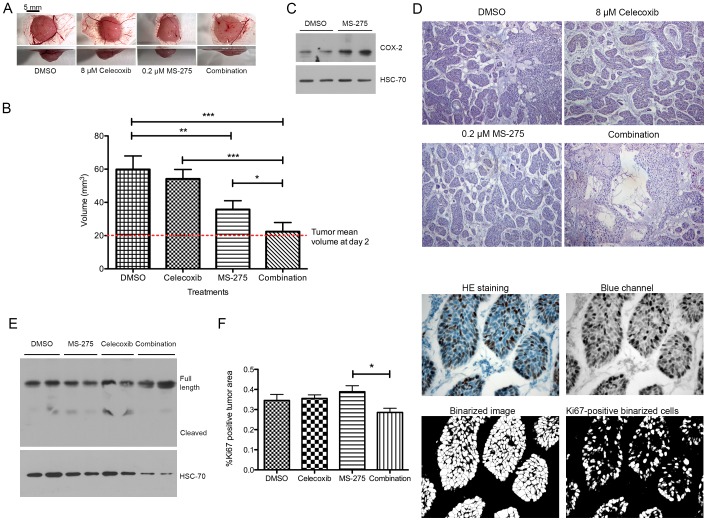
Effect of HDAC and COX-2 co-inhibition on BxPC-3 tumor growth on CAM. (A) Macroscopic pictures were obtained at the same magnification from bottom and side view. (B) Tumor volume at day 7 after cell implantation. Tumors were treated with 30 µl celecoxib (8 µM), MS-275 (0.2 µM) or drug combination at same concentration. (C) Western-blot detection of COX-2 in 20 µg proteins isolated from tumors grown on CAM and treated with MS-275 (0.2 µM). HSC70 was used as a loading control. (D) Histological aspect of tumors grown on CAM during 7 days and treated with 30 µl celecoxib (8 µM), MS-275 (0.2 µM) or drug combination at same concentration. (E) Western-blot detection of caspase-3 in 40 µg proteins isolated from tumors grown on CAM and treated with MS-275 (0.2 µM) or celecoxib (8 µM). HSC70 was used as a loading control. (F) Ki67 immunostaining and associated quantification of tumors grown on CAM during 7 days and treated with 30 µl celecoxib (8 µM), MS-275 (0.2 µM) or drug combination at same concentration. Results are expressed as mean ± s.d. ***P<.001, **P<.01, *P>.05. n≥3 in each condition.

Next, BxPC-3 tumors were treated beginning day 2 either with 8 µM celecoxib or 0.2 µM MS-275 or with a combination of two drugs at their respective concentrations. MS-275 concentration was chosen to fit with the plasmatic concentration measured in Human in a 5 mg/m^2^ weekly dosing schedule [15]. While celecoxib alone did not affect tumor growth, MS-275 alone induced a decreased of tumor growth by 50% (P<.001) and induced the expression of COX-2. Combination of celecoxib and MS-275 completely abolished (P<.001) tumor growth, leading to no change in tumor volume compared to the beginning of treatment (Figure 9A-B). Tumors treated with MS-275 overexpressed COX-2 (Figure 9C). Tumors treated with combination of celecoxib and MS-275 revealed empty spaces inside the tumor. (Figure 9D). We then asked the question whether this reduction of tumor volume is due to induction of apoptosis or to proliferation arrest. Tumors treated with MS-275, celecoxib or both drugs were submitted to a cleaved caspase-3 detection and were labeled for Ki67. The full-length caspase-3 was detected in all samples but no cleaved caspase-3 was observed (Figure 9E). The relative Ki67-positive area was slightly but significantly reduced by the combination of HDAC and COX-2 inhibitors (Figure 9F).

## Discussion

The potential interest of anti-HDAC treatment strategies for PDAC is supported by several preclinical studies [18], [19], [22], [47]–[50]. In agreement with these studies, we showed that pan-HDAC inhibitor SAHA was able to reduce significantly pancreatic cancer cell growth. Following the rationale that HDAC7, HDAC3 and HDAC1 have been reported to be over-expressed in the PDAC [8]–[10] we have examined their individual roles with respect to their ability to control BxPC-3 cell growth. The results demonstrated that HDAC7 silencing was unable to decrease the cell growth while HDAC1 and HDAC3 inhibition or silencing reduced significantly the BxPC-3 cell growth highlighting the importance of these enzymes in PDAC patients. However, the results of clinical studies where HDAC inhibitors are used show only limited or no ability to affect tumor development [3], [13]. This is likely to be related to the pleiotropic activities of HDAC including some that might promote tumor progression. In this line, HDAC1, –2 and –3 may have been shown to regulate the function of RelA/p65 subunits of NF-kB. Class I HDAC1 can indeed interact with RelA/p65 acting as a corepressor to negatively regulate its transcriptional activity [43]. HDAC3-mediated deacetylation of RelA/p65 promotes its binding to IKBα leading to cytosolic sequestration [42] and NF-kB repression. In parallel, HDAC2 was also overexpressed in PDAC and was shown to regulate NF-kB activity without direct interaction with p65 [43]. As a consequence, class I HDAC inhibition could induce the transcriptional activation of NF-kB-driven genes. Consistently, a significant COX-2 induction was recently showed in lung cancer cells following trichostatin A or SAHA treatment [27]. Here, we showed, for the first time, that the class I HDAC chemical inhibitor MS-275 and selective silencing of both HDAC1 and HDAC3 are able to induce the transcription of COX-2 gene and the accumulation of the functional enzyme independently of the KRAS status. Conversely, HDAC2 silencing does not elicit COX-2 accumulation but reduce its expression. COX-2 is considered to be part of the positive feedback loop amplifying *Ras* activity to a pathological level causing inflammation and cancer [51]. Moreover, COX-2 was demonstrated to confer a growth advantage to pancreatic cancer cells [52]. These results together with our findings suggest the potential interest in inhibiting COX-2 activity while subjecting COX-2 positive (about 50-60% of the cases [53]) PDAC patients to anti-HDAC treatments. This can be easily achieved because several molecules, including the celecoxib [54], were developed in order to inhibit specifically COX-2. Celecoxib was found to significantly decrease or delay pancreatic cancer progression in animal model [29], [55]. Keeping these findings in mind, we combined class I HDAC and COX-2 inhibitors and test their efficiency to control tumor growth. The co-treatment reduced the pancreas cancer cell growth by blocking cells in G0/G1 state. This is probably a mechanism that could explain the effects observed *in vivo*, where the combination of two drugs completely stalled the tumor growth. Importantly, the inhibition of tumor growth was observed with drug concentrations 10-fold lower than the concentrations needed if the drugs were used individually [56], [57]. This represents a considerable advantage for a putative clinical use regarding the possible undesired effects. However, the *in vivo* model used in this work remains very simple compared to the complexity of the pathology in human. Moreover, the cell line used to grow the tumor in ovo is a limitation as it does not harbor constitutively active Kras which is the most common genetic alteration in human PDAC. In consequence, *in vivo* studies in genetically-engineered mouse models of PDAC are more than necessary before entering potential clinical trials with combined treatment, especially in the case of patients harboring KRAS mutation. Several models are now available to recapitulate the disease [58].

One additional outcome of the current study is the development and characterization of a refined animal model of PDAC recapitulating all the main features observed in human tumors. We have based our development on a model we previously set-up [32] but which did not provide with the possibility to efficiently test experimental therapies. Following extensive method development we have established means to produce larger tumors, bearing fully functional blood vessels. The clinical relevance of this improved model is supported by the CK7^+^/CK19^+^/CK20^-^/CEA^+^/Ki67^+^/CD56^−^ immunodetection. CK7 and CK20 expression has been shown to be useful in the differential diagnosis of several carcinomas of epithelial origin. According to Lee et al. [59] 95% of PDAC are CK7^+^, 100% are CK19^+^ and 73% are CK20^−^. In pancreas carcinomas the proportion of cells stained for CEA and the Ki-67 index were respectively increased 3-fold and 10-fold in comparison with the normal tissue [60], [61]. CD56 staining was found negative in all cases of human PDAC [62]. These biomarkers, together with the presence of mucin are the main hallmarks of PDAC [63].

Recently, we have discovered several biomarkers of human PDAC that bare therapeutic potential [46]. These antigens were also present in our CAM tumor model, supporting its similarity with human cancer and providing the research community with a rapid and cost effective model for pancreas cancer research such as our present demonstration of the benefit to combine COX-2 and HDAC inhibition for optimal anti tumor activity.
